# From Carbon Monoxide Poisoning to Myocardial Infarction

**DOI:** 10.7759/cureus.51201

**Published:** 2023-12-27

**Authors:** Cristiana Camacho, Fausto S Pinto, Cláudio Gouveia, Beatriz Chambino, Catarina Rodrigues

**Affiliations:** 1 Internal Medicine, Centro Hospitalar de Lisboa Ocidental, Lisbon, PRT

**Keywords:** acute myocardial injury, st-elevation myocardial infarction (stemi), hyperbaric oxygen therapy, poisoning, carbon monoxide

## Abstract

Myocardial injury is a known complication following acute carbon monoxide (CO) poisoning, yet there is little literature on this subject. Here, we present the case of a 56-year-old man admitted to the emergency room with severe CO poisoning. The electrocardiogram (ECG) at admission revealed an ST-segment elevation in leads II, III, and aVF, with an elevated troponin-T level. The patient was initially treated with hyperbaric oxygen, with improvement of symptoms and ECG normalization. He was later admitted for coronary angiography, which revealed an occlusion of the right coronary artery. This case aims to highlight an often-overlooked complication of CO intoxication and the need for more studies to better guide the treatment of these patients.

## Introduction

Unintentional carbon monoxide (CO) poisoning continues to be one of the main causes of poisoning-related death in the United States. Approximately 100,000 people visit the emergency room (ER) annually due to unintentional non-fire-related CO poisoning [1]. The symptoms of CO poisoning are nonspecific and can range from headache and dizziness to coma and death, with a mortality rate of 1-3% [2,3].

Although it can affect all organs and systems, neurological and cardiac effects dominate as they are the most susceptible organs to hypoxia. Myocardial ischemia is a known complication of CO poisoning, occurring with a frequency of 20-37%, and is associated with poorer prognosis [4,5]. However, despite its frequency and contribution to the prognosis, there are few studies on myocardial injury in the context of CO poisoning.

## Case presentation

We report the case of a 56-year-old active smoker with no other medical history. He was admitted to the ER with nausea, vomiting, and mild chest pain that had started three hours before hospital admission. The pain was described as oppressive, had a transient duration, and was absent at the time of arrival. The patient stated that he was exposed at home to an indoor heater gas bottle in a closed room at the time of symptom onset. Other occupants of the same room showed similar complaints and were also brought to the ER. The CO exposure duration was unknown. Upon admission, the patient was conscious, agitated but cooperative, with a non-invasive blood pressure of 156/80 mmHg, regular heart rate of 80 beats/minute, and temperature of 36.8°C. He presented with no respiratory distress, with an oxygen saturation of 98% in room air. Upon suspicion of CO intoxication, a non-rebreathing mask was used with an oxygen flow of 15 L/minute.

**Figure 1:**
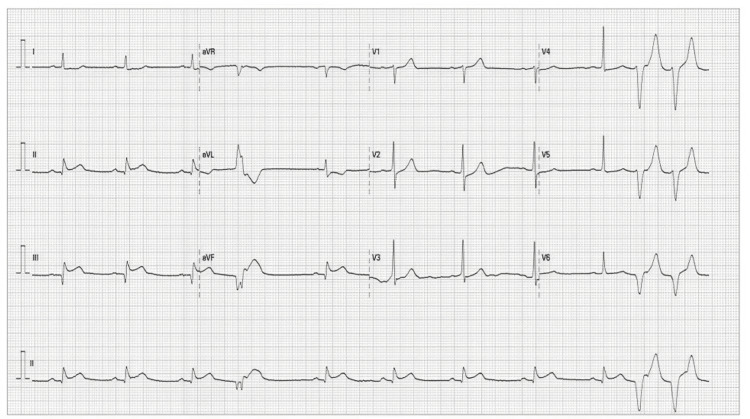
Electrocardiogram at admission showing ST elevation in leads II, III, and aVF.

A blood gas analysis showed a carboxyhemoglobin (COHb) of 26% and lactate of 5.1 mmol/L. The initial electrocardiogram (ECG) revealed an ST elevation in leads II, III, and aVF (Figure 1).

Cardiac enzymes were slightly elevated at admission (troponin T of 34 ng/L). The echocardiogram was normal, with no regional wall motion abnormalities. Considering the absence of pain, mild troponin elevation despite the onset time of pain, and the absence of alterations in the echocardiogram, it was considered that the symptoms and ECG alterations were in the context of CO poisoning. As such, it was decided that the patient should undergo hyperbaric oxygen therapy. He was transferred to a specialized center where he received treatment with hyperbaric oxygen at 2.5 atmospheres absolute for 90 minutes. After treatment, his symptoms resolved, and the ECG went back to normal. Ten days later, he was admitted for further investigation. The cardiac tomography showed evidence of inferior myocardial infarction, 70-99% mixed plaque stenosis of the proximal segment of the right coronary artery (RCA), 50-69% non-calcified plaque stenosis in the mid-RCA, and a 50-69% non-calcified plaque stenosis of the left main coronary artery. The coronary angiography revealed a proximal occlusion of the RCA (type C, moderate-to-severe calcification, with retrograde filling of the distal segment by hetero collaterals) and 30-50% stenosis of the left main coronary artery. Coronary angioplasty with stenting was subsequently performed on the RCA. The patient was later discharged with dual antiplatelet therapy (aspirin and clopidogrel), atorvastatin, bisoprolol, and ramipril, together with a recommendation for smoking cessation. He maintained follow-up Cardiology consultations, remaining asymptomatic six months after the event.

## Discussion

CO toxicity is a result of tissue hypoxia and direct cellular damage [6]. The affinity of CO for hemoglobin is 200-300 times more than that of oxygen. The formation of COHb decreases oxygen transport capacity and leads to a left shift in the oxyhemoglobin dissociation curve, reducing the release of oxygen into the peripheral tissue. CO also leads to a more direct tissue injury with the development of oxygen radicals and inhibition of aerobic metabolism, leading to apoptosis [3,7,8]. CO poisoning can exacerbate myocardial ischemia by breaking the balance between oxygen demand and supply, particularly in individuals with pre-existing coronary artery disease [8,9].

The heart and brain are the organs most affected by CO poisoning due to their higher oxygen demand. Neurological examination may reveal headache, dizziness, confusion, agitation, and, in severe cases, seizure or reduced consciousness [7,10]. Considering that the symptoms are varied and nonspecific and that chest pain may not be present even in the case of myocardial ischemia, it is necessary to perform an ECG and measure cardiac enzymes [11].

Despite the importance of CO measurement for diagnostic confirmation, patients with a suggestive story and symptoms should start treatment as soon as possible. Early administration of 100% oxygen is recommended for all patients in whom this diagnostic suspicion exists [3]. Limited evidence suggests that hyperbaric oxygen results in reduced complications and better outcomes following CO poisoning [10]. Hyperbaric oxygen should at least be considered in all cases of serious acute CO poisoning, namely, patients presenting with COHb levels greater than 25%, loss of consciousness, neurologic deficits, cardiac ischemia, severe metabolic acidosis, or pregnancy [2,10].

The clinical course of our patient was favorable with hyperbaric oxygen therapy, with the improvement of symptoms and normalization of the ECG changes. Despite being initially unknown, the coronary angiography showed extensive coronary disease, probably present at the time of the event. In our case, CO intoxication acted as a trigger for myocardial ischemia in a patient in the setting of coronary artery disease.

## Conclusions

Cardiac involvement in CO intoxication is often underdiagnosed, highlighting the need to raise awareness of this complication and its implications for prognosis. Patients with suspected CO poisoning should be evaluated with an ECG and markers of myocardial necrosis. Despite the frequency of CO poisoning and its prognostic impact, the best approach is yet to be defined. Further research in this area is needed, along with the creation of treatment and follow-up protocols.
